# Mining Health App Data to Find More and Less Successful Weight Loss Subgroups

**DOI:** 10.2196/jmir.5473

**Published:** 2016-06-14

**Authors:** Katrina J Serrano, Mandi Yu, Kisha I Coa, Linda M Collins, Audie A Atienza

**Affiliations:** ^1^ National Cancer Institute Bethesda, MD United States; ^2^ ICF International Rockville, MD United States; ^3^ Pennsylvania State University State College, PA United States

**Keywords:** weight loss, mobile health, mobile app, data mining, classification

## Abstract

**Background:**

More than half of all smartphone app downloads involve weight, diet, and exercise. If successful, these lifestyle apps may have far-reaching effects for disease prevention and health cost-savings, but few researchers have analyzed data from these apps.

**Objective:**

The purposes of this study were to analyze data from a commercial health app (Lose It!) in order to identify successful weight loss subgroups via exploratory analyses and to verify the stability of the results.

**Methods:**

Cross-sectional, de-identified data from Lose It! were analyzed. This dataset (n=12,427,196) was randomly split into 24 subsamples, and this study used 3 subsamples (combined n=972,687). Classification and regression tree methods were used to explore groupings of weight loss with one subsample, with descriptive analyses to examine other group characteristics. Data mining validation methods were conducted with 2 additional subsamples.

**Results:**

In subsample 1, 14.96% of users lost 5% or more of their starting body weight. Classification and regression tree analysis identified 3 distinct subgroups: “the occasional users” had the lowest proportion (4.87%) of individuals who successfully lost weight; “the basic users” had 37.61% weight loss success; and “the power users” achieved the highest percentage of weight loss success at 72.70%. Behavioral factors delineated the subgroups, though app-related behavioral characteristics further distinguished them. Results were replicated in further analyses with separate subsamples.

**Conclusions:**

This study demonstrates that distinct subgroups can be identified in “messy” commercial app data and the identified subgroups can be replicated in independent samples. Behavioral factors and use of custom app features characterized the subgroups. Targeting and tailoring information to particular subgroups could enhance weight loss success. Future studies should replicate data mining analyses to increase methodology rigor.

## Introduction

Smartphone ownership among American adults has increased from 35% in 2011 to 68% in 2015 [1]. This increase has coincided with the proliferation of smartphone apps, and 19% of all app downloads are related to health, with more than half of them involving weight, diet, and exercise [2]. This provides new opportunities to deliver interventions for health behavior change and weight loss in the United States where obesity rates have remained high [3].

Although apps show great promise for helping individuals lose weight and manage lifestyle habits [4-6], evidence to support the impact of commercial apps on health behavior and weight loss is still lacking. This may be due to the lack of evidence-based weight loss principles in currently available apps [7]. But given the popularity of these apps, the potential implications are far-reaching, not only in terms of disease prevention (eg, diabetes, cardiovascular diseases, cancer) but also in cost-savings [8-11].

Data that are collected from commercial health apps are often not collected with scientific research in mind. However, these apps can reach millions of users. If analyzed with rigorous scientific methods, the potentially rich data collected from these apps may offer important insights into how behavior change occurs in naturalistic settings among large segments of the population. Exploratory analyses, such as data mining methods, that can be used to examine existing health data are not new [11-13], but they have rarely been used to examine health data collected from commercial apps.

Furthermore, scientific methods to examine the reliability and robustness of exploratory analyses (ie, data mining validation methods) have also been available for some time [14,15], but have not been used with health app data. With millions of individuals using commercial health apps, opportunities now exist for both exploratory data mining and data mining validation methods to occur in rapid succession. Data mining validation methods increase the scientific rigor of exploratory approaches by testing whether initial findings are stable.

To our knowledge, no studies have explored the effectiveness of a weight loss commercial app AND evaluated the reliability of the exploratory findings. The purposes of this study were to (1) assess the prevalence of weight loss among overweight and obese adults from data gathered by a commercial app, (2) identify successful weight loss subgroups and their characteristics using exploratory data mining techniques, and (3) examine the reliability of the identified subgroups using independent samples.

## Methods

### Dataset

We analyzed a subset of cross-sectional, de-identified data (n=12,427,196), which were obtained directly from Lose It! (FitNow Inc., Boston, MA, USA). Data were made available to researchers at the National Cancer Institute for research purposes only. Lose It!—launched in 2008—is a weight loss app that is available through both iOS and Android app markets, as well as through the Web. Lose It! (henceforth, called the app) provides users with tracking tools (eg, barcode scanners); connections with other devices and apps (eg, Fitbit, RunKeeper); motivation and support (eg, connection with friends); and nutrition feedback (eg, system-generated reports comparing a user’s food log with the US Department of Agriculture’s MyPlate recommendations).

In the app, a user creates an account and a weight loss plan based on one’s height, weight, exercise level, target weight goal, and desired weekly weight loss. The app then uses all this information to calculate an estimated calories budget that is intended to produce the energy deficit required to meet one’s weight loss goal. The weight loss plan consists of logging one’s diet, exercise, and weight through either self-report or a synced device (eg, WiFi-connected body scales). The app offers motivation and support tools by allowing users to identify friends and share progress and information with them. Users can also participate in groups designed to motivate users; for example, one featured group—“We’re all in this together!”—is described as “a group for people looking to give motivation and people looking to get motivation.”

The data analyzed were from users who had the app during the years of 2008-2014. Data provided for analysis were from the app’s metadata reporting database, which is used to power the app and provides a general summary of user activity. Thus, the data analyzed were cross-sectional in nature. The dataset included the following information: age at setup of the account, gender, height, body weight, body mass index (BMI), desired goal weight, desired weekly weight loss, number of days logged in for food and exercise, number of exercise calories burned, number of calories consumed, number of times weighed in, number of days active, date of last activity, devices and apps connected to a user’s account, type of operating system used, number of friends and groups on the app, number of challenges users participated in, number of customized goals, foods, recipes, and exercises users entered, and app-specific options (eg, has a picture, uses reminders). Weight and health behavior data were self-reported, whereas technical-related data (eg, type of operating system used, app-specific options) were from the app’s database. More time-intensive longitudinal data for the full sample of users between 2008 and 2014 were not readily available at the time of analyses.

Data cleaning was required before analyses, which included removing any duplicate records, placing valid ranges for each variable, and distinguishing between missing versus invalid data. There were 63,641 duplicates that were deleted. These users had the exact same information for all weight, health, and technical-related variables. We were left with a total sample of 12,363,555. Analyses with this entire sample proved to be challenging and required more computing memory than typically offered by a single computer. Therefore, for computing management and efficiency, this dataset was randomly split into 24 subsamples, each with a sample size of approximately 500,000. This study used 3 subsamples and excluded the following in each subsample: (1) participants who reported being less than 18 years or greater than 70 years at setup age—older adults (65 years and older) are less likely to use health-related smartphone apps [2], so to be more conservative, we chose 70 years as the upper age range; (2) participants who reported being younger than 18 years at the date of last activity; (3) participants who were underweight and of normal weight, BMI ≤24.9; and (4) participants with weight and weight loss values that were out of range; for example, we defined minimum weight values that exceeded start weight values as out of range.

The outcome of interest was weight loss, defined for the purpose of this study as losing 5% or more of a user’s starting body weight, which has been shown to lead to beneficial health effects [16-18]. This was calculated by subtracting 5% of a user’s starting weight from a user’s minimum weight. If this number was less than or equal to zero, then weight loss was categorized as yes, all others were categorized as no. The following predictors were included in the analyses: age, gender, number of weigh ins, target weight, weekly weight loss goal, start weight, start BMI, food and exercise days logged, average food and exercise calories logged, days active on the app, age at set up of the app, type of device or app used, type of operating system used, number of friends, number of groups, number of challenges, use of reminders, customized goals, customized recipes, customized exercises, and app-specific options.

### Statistical analysis

Classification and regression tree (CART) analysis was conducted in subsample 1 (hereafter, known as the training sample). CART methods have been increasingly applied to health behavior research for exploratory purposes [19-23]. CART analysis is a type of decision tree methodology, also called recursive partitioning, that is useful for constructing prediction models from data [19,20,24-26]. CART uses nonparametric statistics to identify mutually exclusive and exhaustive subgroups of individuals who share common characteristics that influence the dependent variable of interest. The CART procedure uses a preselected splitting criterion to assess all possible independent variables and chooses a variable (ie, splitting variable) that results in binary groups that are the most different with regard to the dependent variable. The splitting criterion used was the Gini index of diversity [25], which selects the split that maximizes the reduction in impurity or diversity of a node, thereby reducing the error in classification [19,25].

CART methods have several advantages over more traditional approaches, such as logistic regression. Because CART is inherently nonparametric, no assumptions are made about the underlying distribution of the data. Thus, it can handle highly skewed distributions or even extreme scores or outliers [19,20,26]. CART also has sophisticated methods for handling missing data, and missing data are considered for each variable at each split point. If data are missing at a particular split point, surrogate variables that contain similar information to the primary splitter are used [27,28]. This is also an important consideration given the missing data typically seen in commercial health app data.

The CART analysis was conducted in R (version 3.1.3), using the package rpart. The default settings for rpart were used, and these parameters have been recommended by Breiman and colleagues [25]. More details about this package are provided elsewhere [28]. We then created mutually exclusive subgroups in the training sample based on the CART results. Descriptive analyses were conducted in SAS (version 9.3, SAS Institute, Inc., Cary, NC, USA) with the training sample to determine whether additional factors were uniquely associated with the various subgroups. Due to the large sample size, we were dubious of interpreting the *P* values; therefore, significance was determined by the unique variance explained by the predictor variables (using *R*^2^ or Cramer’s V). As a rule of thumb, the proportion of variance accounted for by the predictor variable had to be at least 1%.

The CART model predictions identified from the training sample were then evaluated with subsample 2 (hereafter, known as data mining validation sample 1) to examine the robustness of the model. The area under the receiver-operating characteristic curve (AUC) was used to evaluate the accuracy of the classification tree with data mining validation sample 1. Further evaluation was conducted with subsample 3 (hereafter, known as data mining validation sample 2), and the AUC was also obtained with this subsample. The AUC analyses were conducted in R (version 3.1.3), using the package pROC. More details about this package are provided elsewhere [29]. The annotated code regarding these analyses can be found here: https://github.com/kayserra/sample_code. For exploratory purposes, we also applied CART methods with data mining validation sample 2. We varied the default settings for the complexity parameter (ie, a criterion that takes into account the consequences of misclassification) to 0.001 versus 0.01 and the minimum number of observations in a node to compute a split as well as the terminal node to 3000 (1% of the sample) versus the default of 20 and 7, respectively.

## Results

### Analytic sample

**Figure 1:**
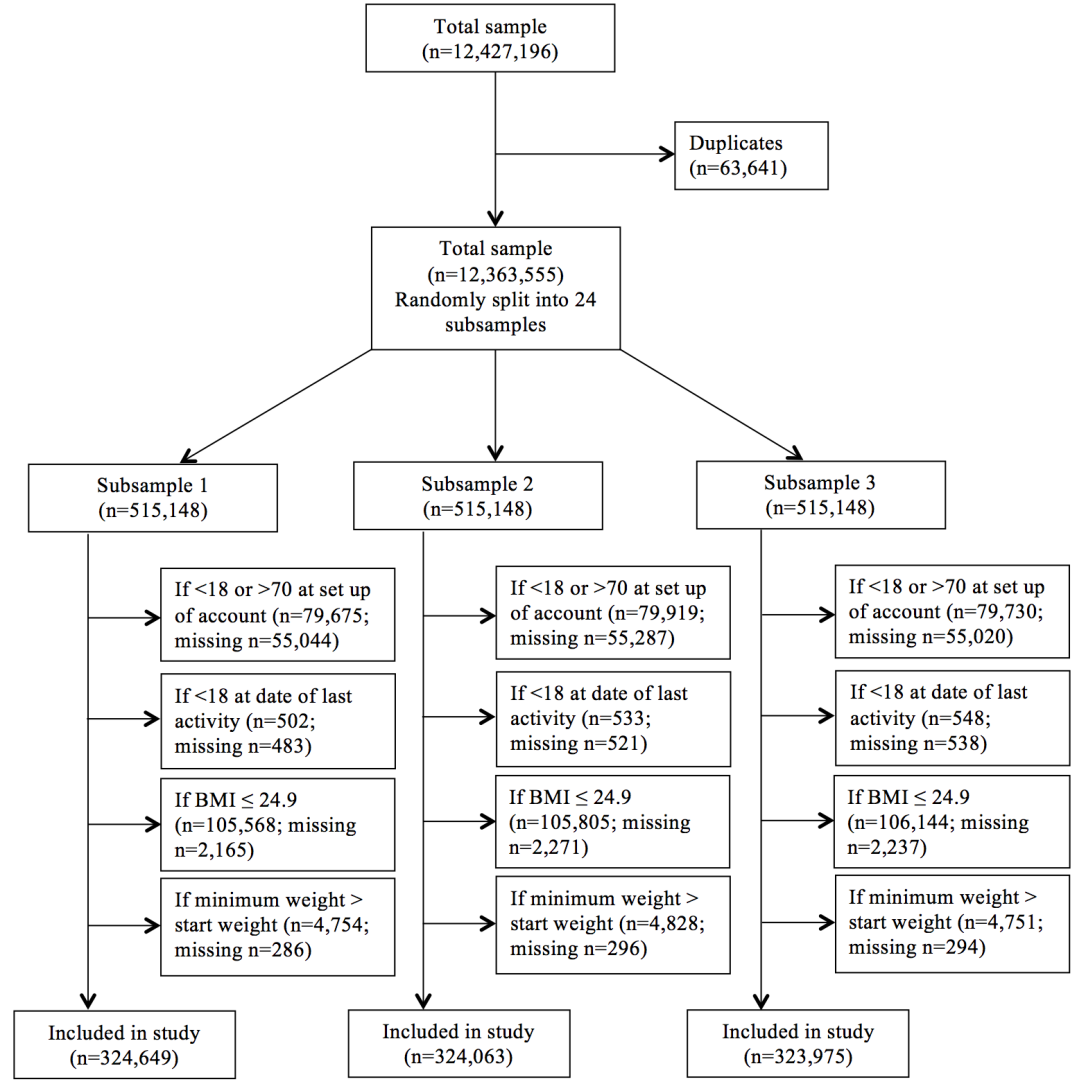
Data flow chart.

Data cleaning and exclusion criteria applied to the 3 subsamples resulted in the following analytic samples: n=324,649 for subsample 1, n=324,063 for subsample 2, and n=323,975 for subsample 3 (data flow chart shown in Figure 1).

### Statistical analysis

**Figure 2:**
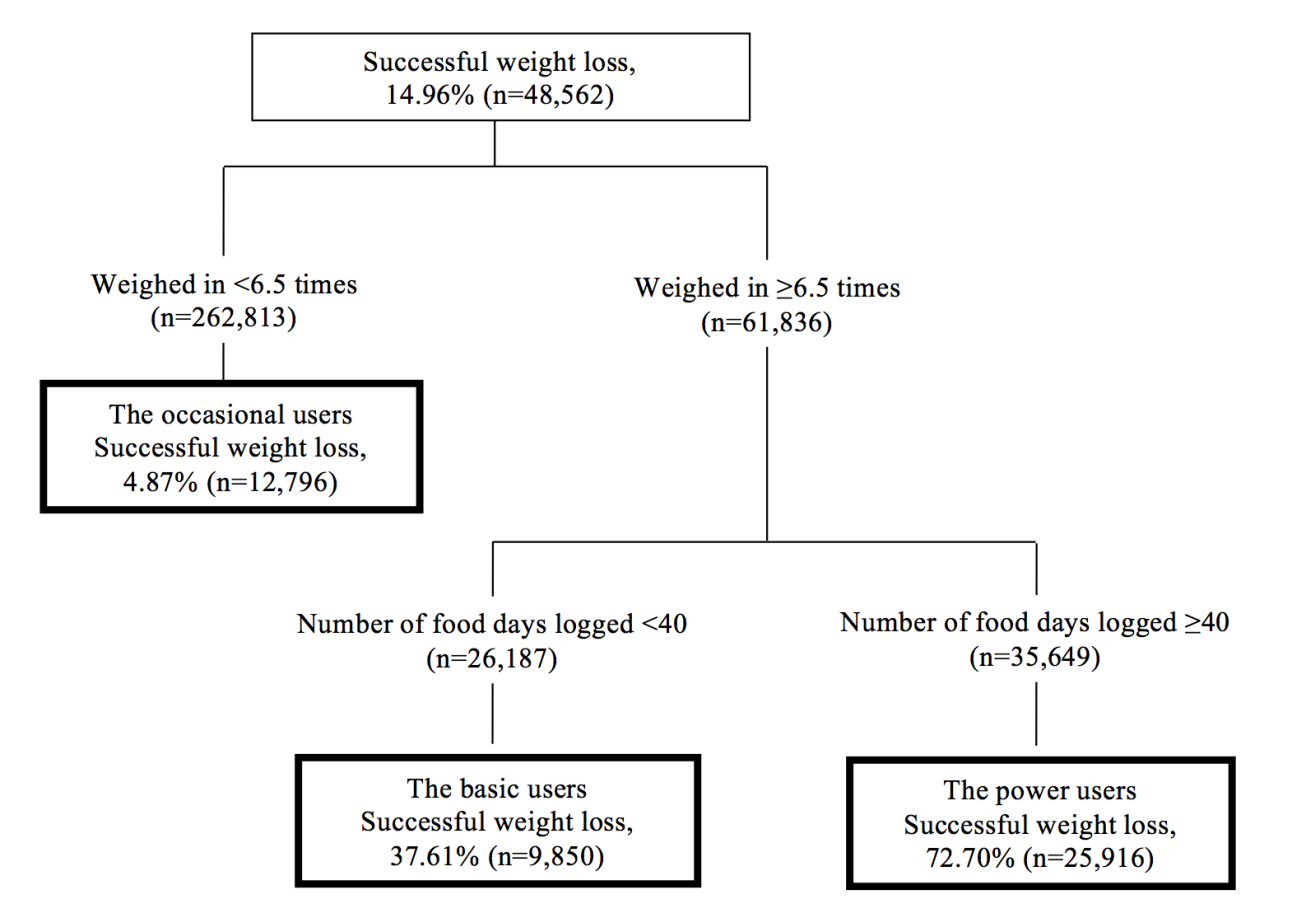
Classification and regression tree for identifying successful weight loss subgroups with the training sample (n=324,649).

The CART model is displayed in Figure 2. As shown in the figure, 14.96% (48,562) of the training sample successfully lost weight. The CART analysis identified 3 distinct subgroups that we labeled for descriptive purposes: “the occasional users,” “the basic users,” and “the power users.”

Although descriptive names are given for each subgroup, to more fully understand and interpret the subgroups, a set of additional characteristics were further examined. Results for the descriptive analyses that examined additional unique characteristics among the subgroups are displayed in Table 1.

**Table 1:** Additional characteristics of identified successful weight loss subgroups with the training sample (n=324,649).

		The occasional users^a^	The basic users^b^	The power users^c^	Cramer’s V	R^2^	*P*
		% or mean (standard deviation)	% or mean (standard deviation)	% or mean (standard deviation)			
**Demographics**							
	Female	74.32%	71.68%	63.53%	0.1049		<.001
	Age (at set up of account)	34.5 (12.0)	35.4 (11.3)	39.0 (12.1)		0.0297	<.001
	Start weight	212.0 (50.8)	211.3 (47.3)	211.2 (47.4)		0.0001	.274
	Start BMI^d^	33.9 (7.0)	33.6 (6.6)	33.0 (6.4)		0.0034	<.001
	Days active on the app	23.5 (46.0)	21.9 (10.4)	168.3 (174.7)		0.2383	<.001
**Health behaviors**							
	Exercise days logged	9.8 (29.0)	9.0 (7.7)	80.5 (112.7)		0.1522	<.001
	Exercise calories logged	39081969.7 (2931936775.4)	3799.2 (4979.1)	7753953.9 (1242356057.6)		0.0001	.169
	Food calories logged	7844318.9 (884021907.5)	1040215.2 (100758647.6)	11818596.1 (1075871163.0)		0.0000	.602
	Goal weight	160.5 (33.4)	161.8 (32.4)	166.2 (32.7)		0.0062	<.001
	Goal plan^e^	1.7 (0.4)	1.8 (0.4)	1.6 (0.5)		0.0139	<.001
**App behaviors**							
	iPhone users (% yes)^f^	71.59%	72.84%	77.73%	0.0653		<.001
	Android users (% yes)^f^	29.40%	31.60%	30.88%	0.0171		<.001
	Web users (% yes)^f^	4.01%	3.84%	2.94%	0.0277		<.001
	One or more devices/apps linked with app (eg, Fitbit) (% yes)	3.70%	7.82%	14.00%	0.1487		<.001
	Has friends on the app (% yes)	18.01%	27.37%	43.44%	0.2356		<.001
	Number of friends on the app	0.3 (1.3)	0.6 (2.2)	2.1 (14.4)		0.0062	<.001
	Is part of a group on the app (% yes)	1.41%	3.21%	5.45%	0.0894		<.001
	Number of groups on the app	0.0 (0.3)	0.1 (0.4)	0.1 (1.1)		0.0039	<.001
	Has been an administrator of a challenge (% yes)	0.02%	0.05%	0.32%	0.0332		<.001
	Number of challenges participated in	0.0 (0.0)	0.0 (0.0)	0.0 (0.2)		0.0007	<.001
	Number of customized goals entered	0.0 (0.4)	0.1 (0.7)	0.3 (1.4)		0.0127	<.001
	Number of customized foods entered	5.9 (16.6)	7.7 (13.3)	43.9 (81.4)		0.0866	<.001
	Number of customized recipes entered	0.4 (2.2)	0.5 (2.0)	4.3 (13.4)		0.0373	<.001
	Number of customized exercises entered	0.5 (8.0)	0.6 (2.7)	3.1 (19.9)		0.0071	<.001
	Uses app reminders (% yes)	5.97%	8.30%	14.23%	0.1189		<.001
	Has a picture (% yes)	9.60%	15.54%	25.70%	0.1780		<.001
	Uses email reports (% yes)	1.45%	2.88%	6.22%	0.1048		<.001

^a^4.87% achieved weight loss success (n=12,796).

^b^37.61% achieved weight loss success (n=9,850).

^c^72.70% achieved weight loss success (n=25,916).

^d^BMI, body mass index.

^e^Desired weekly weight loss (0-2 lbs).

^f^Users can download and access the app on multiple platforms and devices.

The occasional users achieved the lowest percentage of weight loss success (4.87%), and these users weighed in on the app <6.5 times. Approximately 37.61% of the basic users achieved at least 5% weight loss, and these individuals weighed in at least 6.5 times and logged in food <40 days. The power users had the highest percentage of weight loss success (72.70%) and consisted of individuals who weighed in at least 6.5 times and logged in food ≥40 days.

Compared with the other subgroups identified, the power users had more men (36.47%) than the occasional users or the basic users, and they were more active with the app (about 168 days). They also logged in more days of exercise. The majority (77.73%) of the power users used an iPhone versus Android, and a lower percentage were Web users as compared with the occasional or basic users. A higher proportion also (14.00%) had at least one or more devices/apps linked to the app versus the occasional users (3.70%) or the basic users (7.82%). The power users also had more friends on the app; were part of a group; had been an administrator of a challenge; and had more customized goals, foods, recipes, and exercises than the other subgroups. They also had a higher percentage of app customization (eg, app reminders, setting up a picture).

**Figure 3:**
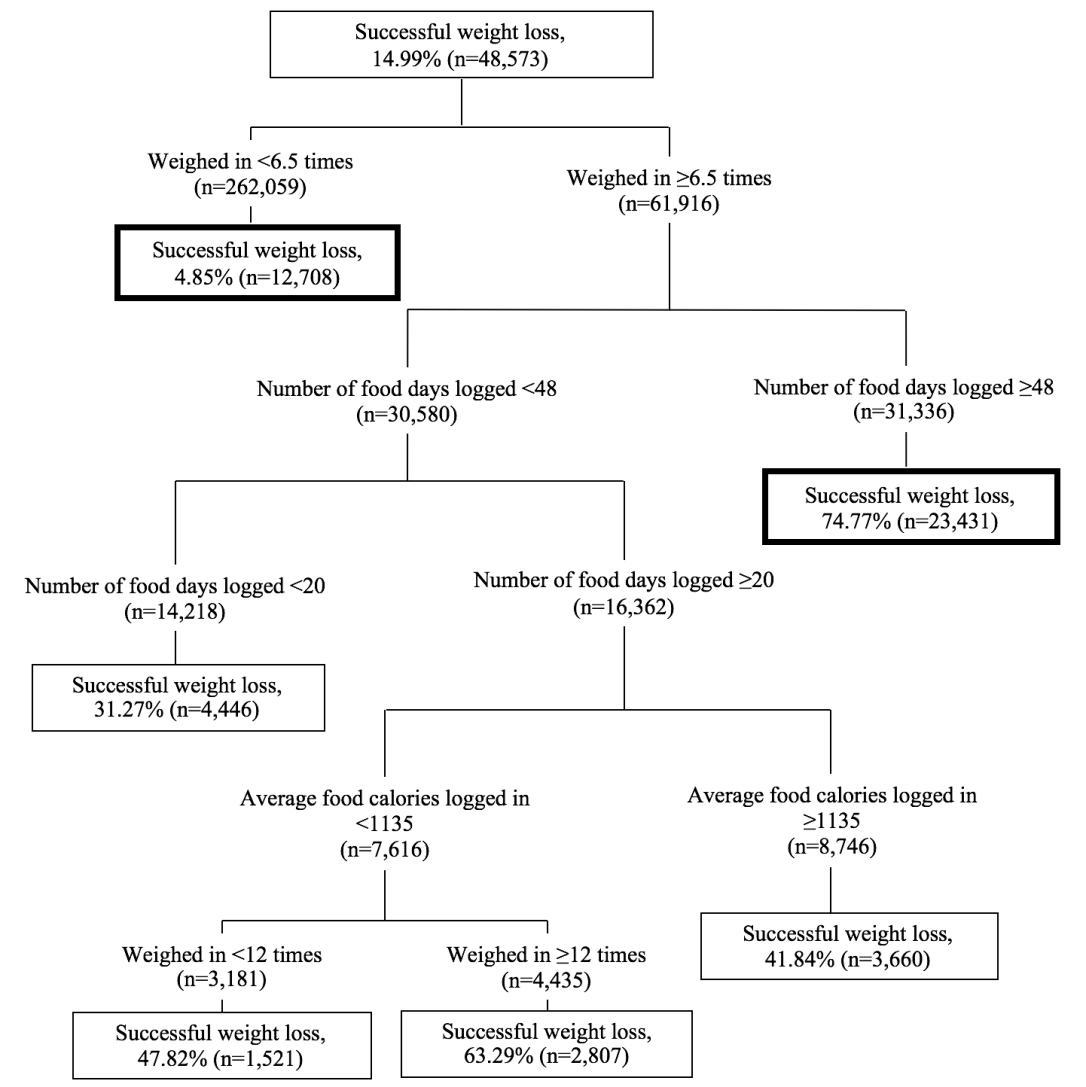
Classification and regression tree for identifying successful weight loss subgroups with data mining validation sample 2 (n=323,975), varying the complexity parameter, minimum node split, and terminal node. Note: Factors for initial splits are similar to Figure 2. Subgroups from similar splits are bolded.

With respect to the robustness of the exploratory analyses, the AUC obtained from data mining validation sample 1 was 0.8327 (95% CI, 0.8306-0.8348), indicating good accuracy. The AUC obtained from data mining validation sample 2 was 0.8339 (95% CI, 0.8318-0.8359); thus, indicating high reliability. The CART model using data mining validation sample 2 is shown in Figure 3.

The factors used to predict the initial splits were almost identical to the model obtained from the training sample. Varying the complexity parameter in data mining validation sample 2 further subdivided the weight loss subgroups, based on food calories logged and weigh-ins. The overall model, however, is comparable to the initial model that used the training sample.

Based on the results that characterized the subgroups identified in the CART analyses, customization of the app appeared to be important among those who were more successful at losing weight. The group with a higher proportion of weight loss (the power users) used more features of the app than the other 2 weight loss subgroups. To explore the extent to which customization led to higher weight loss success, we conducted a logistic regression analysis post hoc using the training sample, with weight loss as the outcome and customization as the predictor. Weight loss was treated the same way as aforementioned, a dichotomous variable representing 5% or more of user’s starting weight, and customization was derived as an ordinal variable consisting of 5 values (0-4 or more) that represented the number of customization features a user had (ie, whether a user had friends; was part of a group; administered a challenge; had custom goals, exercises, foods, or recipes; used reminders; used email reports; or had a picture). The odds of weight loss success progressively increased with more customization features compared with no customization features (1 customization feature: odds ratio, OR=5.27, 95% CI=5.11-5.44; 2 customization features: OR=12.39, 95% CI=11.99-12.81; 3 customization features: OR=22.42, 95% CI=21.56-23.31; 4 customization features: OR=48.30, 95% CI=46.23-50.46). Similar results were obtained with data mining validation sample 1.

## Discussion

Commercial weight loss apps can reach large segments of society, and data from these apps can provide possible clues to subgroups that are more or less successful at losing weight. However, these data can be messy and few researchers have attempted to systematically detect the signal from the noise with this type of data, using exploratory data mining methods. In addition to providing a model for exploring large quantities of commercially generated mobile health data, this study used analytic techniques to systematically examine the robustness and reliability of results obtained from exploratory analyses.

Results indicated key behavioral factors (eg, the number of times a user weighs in and the number of food days a user logs on the app) classified subgroups with varying proportions of weight loss success. On further exploration of characteristics of weight loss, users who were more successful at weight loss logged in about 8 times more days of exercise than the other subgroups. These findings are consistent with the literature demonstrating frequent self-monitoring, such as weighing in and logging in food and exercise, is associated with greater weight loss and decreased risk of weight regain [30-34].

Unexpectedly, this study found that the most successful weight loss subgroup (the power users) had a significantly higher number of iPhone users, compared with Android or Web users. Whether this is due to differences in iPhone versus other users or differences in the user experience of the app is unclear. Moreover, having friends on the app appears to be an important characteristic of weight loss, accounting for about 24% of the variance between subgroups. The power users had about 25% more friends on the app than the occasional users. Studies have shown that social networks have become commonplace for individuals wanting to share information and seeking emotional support for issues regarding weight loss [35,36], and this is highly correlated with weight loss [37-40].

This study further suggests that greater customization of the app is associated with more likelihood of successful weight loss. Thus, although key behavioral factors are important in identifying more versus less successful weight loss subgroups, how users interact with the app may also be important. It may be possible that individuals who customize their app tend to be more engaged with their app, and those who are more engaged are more likely to be more motivated. This hypothesis warrants further investigation.

### Limitations

There were a number of limitations associated with this study. First, the sample may not be representative of a national population. To examine this, we compared our entire app sample with a nationally representative sample (ie, 2008-2014 National Health Information Survey, NHIS, data) to examine differences. When we restricted both samples to include those aged only 18-70 years old (the app: n=10,444,981; NHIS: n=186,134 with replicate weights), we found that the app sample had a higher percentage of women (75.40%) than the NHIS sample (50.96%). The app sample was slightly younger (35.5 years) than the NHIS sample (42.6 years). When we applied both age and weight exclusion criteria to include only overweight and obese adults, these differences persisted, although the average BMIs were comparable between the 2 samples.

Second, the weight data were self-reported which may lead to inaccurate data. We examined the BMI values in the app sample with the NHIS sample where BMI is also calculated using self-reported data. The NHIS sample had a lower average BMI, 27.8, compared with the app sample where the average starting BMI was 30.4. When we examined only overweight and obese adults, the app sample had only slightly higher starting BMI values than the NHIS sample (32.8 vs 31.0). Still, whether the results from this study can generalize to overweight and obese individuals more broadly is unknown.

Third, the data we analyzed were metadata and summary data. Therefore, we could only assess changes in weight at a general level, but not more specific longitudinal patterns. Thus, we could not assess more time-intensive longitudinal patterns of weight loss.

### Conclusions

This study provides an approach to apply scientific methods to large health datasets collected by commercial apps and other health behavior technologies. Using both exploratory data mining and validation methods with big data in rapid fashion can increase confidence in the results that are obtained. Researchers should look to optimize scientific rigor, especially when trying to detect signal from noise in messy datasets.

In addition, the identification of particular subgroups that are successful at weight loss may help to inform researchers and practitioners involved in designing interventions with mobile technologies and smartphone apps. For example, weight loss interventions that use mobile technologies might aim to design interventions that emphasize behavioral factors and encourage individuals to customize their app experience. Furthermore, this study used data mining techniques that aid in hypothesis generation. Future studies should test the mechanisms underlying the behavior change, in this case, weight loss.

As more and more health app data become available, methods to analyze such big data will be crucial. Indeed, the era of big data offers new opportunities to better understand health behavior and behavior change, as well as potentially advance health behavior theories that help to explain mechanisms of behavior change. Our study provides an example for researchers to take full advantage of such opportunities.

## Abbreviations

BMI: body mass index
CART: Classification and regression tree
NHIS: National Health Information Survey
